# Circular Intensity Differential Scattering for Label-Free Chromatin Characterization: A Review for Optical Microscopy

**DOI:** 10.3390/polym12102428

**Published:** 2020-10-21

**Authors:** Aymeric Le Gratiet, Riccardo Marongiu, Alberto Diaspro

**Affiliations:** 1 Nanoscopy and NIC@IIT, Istituto Italiano di Tecnologia, Via Enrico Melen 83, 16152 Genova, Italy; riccardo.marongiu@iit.it (R.M.); alberto.diaspro@iit.it (A.D.); 2 Department of Physics, University of Genoa, Via Dodecaneso 33, 16146 Genova, Italy

**Keywords:** biopolymers, polarization, microscopy, DNA organization

## Abstract

Circular Intensity Differential Scattering (CIDS) provides a differential measurement of the circular right and left polarized light and has been proven to be a gold standard label-free technique to study the molecular conformation of complex biopolymers, such as chromatin. In early works, it has been shown that the scattering component of the CIDS signal gives information from the long-range chiral organization on a scale down to 1/10th–1/20th of the excitation wavelength, leading to information related to the structure and orientation of biopolymers in situ at the nanoscale. In this paper, we review the typical methods and technologies employed for measuring this signal coming from complex macro-molecules ordering. Additionally, we include a general description of the experimental architectures employed for spectroscopic CIDS measurements, angular or spectral, and of the most recent advances in the field of optical imaging microscopy, allowing a visualization of the chromatin organization in situ.

## 1. Introduction

Chromatin is a complex chiral macromolecule composed by the DNA double helix and proteins located in the nucleus of eukaryotic cells [1]. The understanding of its different compaction levels is key for describing DNA replication and other nuclear processes that play a primary role in DNA packaging and gene regulation in the cell cycle [2]. The widely used approaches to apprehend its organization are super-resolution optical microscopy [3] and cryo-electron microscopy [4]. The main advantage of optical super-resolution techniques is to allow the detection of localized molecules of interest thanks to specific labeling. However, these methods require chromatin-specific fluorescent probes for labeling, which can interfere with the natural bio-molecular processes. Additionally, the preparation of such samples is a mandatory and time-consuming step that depends on the use of a particular labeling protocol. Label-free microscopy approaches can overcome these issues with high contrast coming from the particular optical properties of the sample.

Among these techniques, Mueller matrix polarimetry provides a complete description of the optical properties of the sample in a non-invasive and label-free way. The light/matter interaction can be summarized in a single 4 × 4 Mueller matrix composed of 16 real elements [5] presented as:

(1) $$\left[M\right]=\left[\begin{array}{cccc} m_{00} & m_{01} & m_{02} & m_{03}\\ m_{10} & m_{11} & m_{12} & m_{13}\\ m_{20} & m_{21} & m_{22} & m_{23}\\ m_{30} & m_{31} & m_{32} & m_{33} \end{array}\right]$$

The mathematical decomposition of this matrix leads to numerous polarimetric parameters, such as the absorption/scattering, Linear/Circular Dichroism (LD/CD), Linear/Circular Birefringence (LB/CB), and the orientation [6]. Over the last few decades, this technique has shown its efficiency in multiple areas, thanks to the sensitivity to the sample conformation [7]. For instance, numerous works have been mostly dedicated to characterizing material media (thin film, dielectric materials, metasurfaces, etc.), converting experimental polarimeters into ellipsometers [8,9,10]. Advances in the remote sensing research field have allowed Mueller polarimeters to focus on the target detection of micro-/nano-particles in a turbid or highly scattering medium [11,12,13,14,15]. The main recent advances have been focused on investigating the modification of the tissues induced by pathologies at the cellular level [16,17,18,19,20,21,22] for biomedical diagnosis and on developing innovative optical label-free polarimetric microscopes [23,24,25].

The physical model to quantify all the polarimetric effects characterizing the structural organization of the non-labeled sample is of particular interest, and it is achieved by modeling the elementary polarimetric arrangement of the Mueller Matrix (MM) as proposed by the gold standard method of the Lu and Chipman decomposition [26]. However, interpreting the full Mueller matrix of random complex biological media at this scale could be an arduous task. The main reason is that extracting the localized polarimetric signature of a biological medium is difficult due to the extremely low Signal-to-Noise Ratio (SNR) and the mix of numerous different structures confined in a small volume. It is the main reason why applications of this technique are widely dedicated to arranged and patterned inorganic material media.

To overcome this limitation, early works have put more emphasis on the study of only a few elements of the MM, more sensitive to particular configurations of the medium. This is typically the case of Circular Dichroism (CD), defined using the intensities of the circular left and right polarization of the light scattered from the sample, arising from media exhibiting an optical activity. In this review, these media are chiral, meaning their structures are similar, but are not the same as their mirrored image, corresponding to completely different physical/chemical properties according to the handedness of the molecule. CD corresponds to the $m_{03}$ (also refereed to as $S_{14}$) element of the 4 × 4 scattering Mueller matrix [27]. It has been proven that the total CD signal comes mainly from absorption, but also a weak scattering component and carries structural information at the single molecule level [28,29,30]. The scattering component of CD, named CIDS (Circular Intensity Differential Scattering) [31], is particularly important outside the absorption band of the sample and/or at a non-zero scattering angle. It originates from long-range chiral structures at a scale of down to 1/20th the wavelength of incident light [32,33] and is sensitive to (1) the characteristics of the chirality, such as their radius and pitch, (2) the handedness (left or right) of the molecules, and (3) the compaction of the chiral groups [34,35,36,37]. CIDS emission is angularly dependent and is described by the following equation:

(2) $$CIDS\left(\theta \right)=\frac{I_{L}\left(\theta \right)-I_{R}\left(\theta \right)}{I_{L}\left(\theta \right)+I_{R}\left(\theta \right)}$$

where $I_{L}$ and $I_{R}$ are respectively the detected intensities for the left and right circular polarization states and θ is the scattering angle.

As previously stated, CD/CIDS and polarization in general can be of interest as label-free techniques to study the structure of biopolymers such as chromatin. The experimental procedure to evaluate the CD sign and magnitude is based on the Mueller/Stokes formalism. It consists of generating and analyzing a set of at least 16 equations of polarization states coming from the interaction between the polarized light and the sample [38]. For typical CIDS measurements, the generation of the circular right and left polarization states is performed through a birefringent crystal stressed periodically using electro-optics devices [39]. Using the Stokes–Mueller formalism combined with a lock-in detection at the reference frequency, the acquisition leads to a single differential signal after interaction in the excitation volume. This results in a drastic simplification of the experimental setup and a higher SNR. By adding multiple optical modulator devices in the setup, it is possible to achieve very short acquisition times for detecting ultra-fast and sensitive changes in the polymer organization [40]. The interesting characteristic of this method is its versatility to be shifted from the simple single point measurement to the 2D imaging acquisition through a microscopy approach without the need for numerous additional optical changes in the polarimetric pathway. The most common application of the CIDS is the single point measurement, in which the scattering from the whole illuminated sample volume is measured either as a function of the wavelength [41] or the scattering angle [42]. This has been proven to be very fitting for virus and bacteria detection and chiral structures’ characterization, justifying the important rise of numerous commercial systems [43].

This review article is dedicated in particular to the description of the experimental advances in the field of CD/CIDS spectroscopy, demonstrating the capability of this method to describe biopolymers’ organization at the nanoscale. For instance, we present briefly studies performed on biopolymers and micro-organisms (viruses, chromatin) for in vitro and in situ characterization. We used databases such as PubMed and the Web of Science to gather information for this article. First, we discuss the different optical methods to extract this signal in the low SNR scattering regime from the literature. Furthermore, we will present the experimental single point measurements and their applications in the field of virus/bacteria detection and biopolymer structure characterization. Finally, we show the capability of this technique to be incorporated in an imaging configuration, demonstrated from early works to the most recent advances in the optical scanning microscopy field for chromatin imaging.

## 2. CIDS Effect

Between the 1970s and the 2000s, a considerable effort using CIDS detection has contributed to the understanding of the molecular conformation of DNA in chromosomes [44,45] or the structure of viruses [46]. In early works, it was proven that the difference in the total scattering cross-section for the circularly polarized light can also contribute to an additional CD effect [44]. CD spectroscopy was already a gold standard technique for studying chiral molecules [47], and propositions for the usage of CIDS as a probe of higher order chiral organization found in the chromatin structures and that of other protein-nucleic acid complexes were quickly advanced [48]. Indeed, the typical CD approach is sensitive to the molecular order of a few angstroms, and it has been shown that CIDS can be sensitive to structures of a size down to 1/20th the size of the incident wavelength, possibly unlocking information normally not available with a conventionnal optical microscope [32]. It is worth noting that CIDS can occur at any wavelength, but is usually studied at wavelengths outside the absorption bands and at angles different from $0^{\circ }$, since the scattering component (CIDS) is usually much weaker than the absorption component [49].

In single point experiments on a turbid medium, the transmitted intensity magnitude is defined by the Beer–Lambert law. The light is modeled as passing through a sample with concentration *c* and path length *l*. We can define the molecular cross-section ϵ (also referred to as the extinction coefficient) as the integral all over the cross-section σ, and it corresponds to the intensity scattered by a molecule at any angle θ. Thus, the CIDS signal can be calculated with the formula [32]:

(3) $$\frac{I_{L}\left(\theta \right)-I_{R}\left(\theta \right)}{I_{L}\left(\theta \right)+I_{R}\left(\theta \right)}=\frac{-2.303}{2}.\left(\epsilon_{L}-\epsilon_{R}\right).c.l.\left[\frac{{\left(1+cos\theta \right)}^{2}}{2(1+cos^{2}\theta )}\right]+\frac{\sigma_{L}\left(\theta \right)-\sigma_{R}\left(\theta \right)}{\sigma_{L}\left(\theta \right)+\sigma_{R}\left(\theta \right)}$$

This expression corresponds to the CIDS at any angle except zero, where only the scattered beam is detected. The first part is related to the differential attenuation of the CD in the absorbed and scattered beam through the cell. The second part corresponds to the CIDS cross-sections at any angle θ. It is worth noting that the magnitude and the sign of the differential scattering signal are not simply related to the cross-section. Indeed, they depend on (i) the relative orientation, (ii) the distances between the chiral groups, (iii) the chirality, and (iv) the size of the scattering particle. The main contribution of the scattering signal is when the distances between the scattering groups vary from 1/20th to one times the excitation wavelength. Additionally, the CIDS magnitude becomes larger when the dimension of the scatterers is of the order of the incident wavelength [32]. Secondly, early works predicted that the left-handed chirality of a macromolecule gives a positive CIDS [31]. Conversely, a right-handed helix will give negative CIDS at a wavelength higher than the dimension of the pitch of the helix [50].

Therefore, this approach has been the subject of numerous theoretical studies to explain the origin of the CIDS signal [51] and quantify the differential scattering cross-section [52]. One of the first approximation methods to model the CIDS signal deriving from chromatin structures was the Born approximation [53], which assumes all scatterers as ideal and independent. However, this approximation does not consider the internal interactions in the scatterer and incorporates anisotropy geometrically by replacing isotropic scatters such as spheres with ellipsoids. For the chromatin structure, we cannot ignore the internal interactions between nucleosomes, which affect the compaction of the chromatin structure [54]. Another approach widely employed is the discrete dipole approximation [55,56], the ADDAcode [57], to compute the Mueller matrix elements. This method discretizes the scattering object into a collection of polarizable point dipoles and takes into account their interaction. The minimum requirement of dipoles to approximate the scattering object is dictated by its refractive index. The polarizability of point dipoles has been calculated using the lattice dispersion relation (LDR) [58] and has been measured in the X-ray region with good agreements [59]. The theory to calculate Mueller matrix elements for a set of scattering angles was adopted from Bohren et al. [27]. To take into account the random 3D spatial distribution in all the scattering volume, an orientation averaging has been performed over Euler angles. The DDA has been proven to be a numerically exact method [60] and has been employed in various research fields from biophotonics [61,62,63] to plasmonics [58,64].

After the 2000s, only a few studies for the double DNA helix using the CIDS have been proposed. In the literature, a few work focused on highlighting the wavelength dependence of the polarimetric response of solid materials and chiral molecules in turbid media [65,66,67].

## 3. CIDS Experimental Architecture

**Figure 1:**
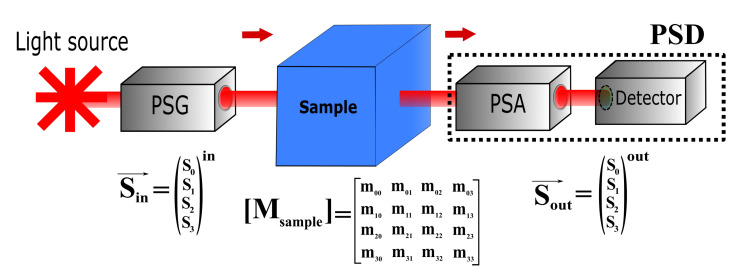
Block diagram of a general MM polarimeter. PSG: Polarization States Generator. PSA: Polarization States Analyzer. PSD: Polarization States Detector. ${\vec{S}}_{in}$ and ${\vec{S}}_{out}$ are the input and output polarization states described by the Stokes vectors.

A general Mueller polarimeter can be described as a successive assembly of multiple optical parts. As presented in Figure 1, it is composed of a light source, an optical block to generate the polarization states called the PSG (Polarization States Generator), the studied sample, another optical block to analyze the transformation of the polarization states after interaction with the medium, called the PSA (Polarization States Analyzer), and the photodetector [68]. In the literature, the mix of the PSA and the detector part is referred to as the Polarization States Detector (PSD).

More specifically, the PSG has the role of encoding the incoming light radiation as described by the Stokes vector $\vec{S_{in}}$ = ${\left[S0,S1,S2,S3\right]}^{in}$. Each Stokes parameter has a physical significance linked to polarized light intensities, and their combinations provide valuable information through various polarization parameters, including the Degree Of Polarization (DOP), Linear (DOLP), and Circular (DOCP) [38]. The polarized light encoding can be made through active rotated polarizing optics such as Linear Polarizers (LP) and waveplates such as Half-Waveplates (HWP) and Quarter-Waveplates (QWP). The drawback of using rotors is mainly the time-consuming operation for generating the polarization states. For this reason, modern CD experiment approaches have proposed to convert the linear polarized light into circular, through passive resonant devices such as Pockels Cells (PC) and Photoelastic Modulators (PEM) [69,70,71]. This technology is composed of a passive crystal subjected to periodic mechanical stress, providing a time-varying birefringence due to the photoelastic effect [39,72]. Thereby, the device can produce circular polarization states with alternating handedness at a rate ranging from tens of kHz to tens of MHz depending on the device used. The most common applications have used the PEM technology despite the higher modulation rate reached by the PC. The reason is that the PC generally is more unstable in time and needs precise feedback control due to a larger strain and optical distortion [73]. Moreover, the PEM has a lot of advantages such as a high optical quality (high power handling capability, large acceptance angle, and large useful aperture), modulation purity and efficiency, and high retardation stability, making it an effective instrument in a wide variety of applications. In Table 1, we resume the different performances of each optical configuration.

After interaction with the sample, the PSA analyzes the transformation of the polarized light, giving the output Stokes vector as $\vec{S_{out}}$ = ${\left[S0,S1,S2,S3\right]}^{out}$, and the light intensity is captured by a polarization-insensitive detector. The scattering signal is extremely weak compared to the directly transmitted intensities, with an intensity ratio of around $10^{-4}$. Despite this, it can be measured using a tunable gain photomultiplier detector. The Mueller matrix, M, describes how the polarization state of input light changes upon interaction with the sample by:

(4) $$\vec{S_{out}}=\left[M\right]\vec{S_{in}}$$

where $\vec{S_{in}}$ and $\vec{S_{out}}$ are the input and output Stokes vectors. Because of the time-variation of the intensity induced by the photoelastic effect, the detected signal is a channeled spectrum represented in the Fourier domain by complex modulation amplitudes at numerous frequencies. Their expression is a linear combination of the MM elements [74,75,76].

**Figure 2:**
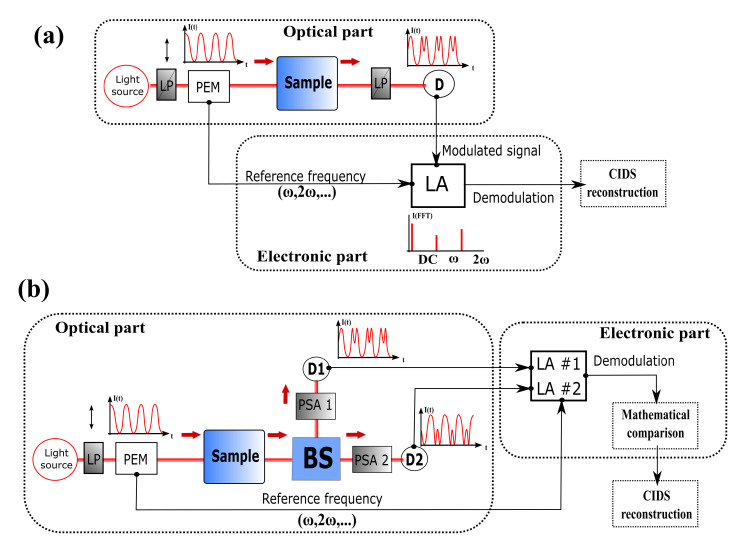
Block diagram of general CIDS setups using one PEM in the PSG (**a**) with a single detector and (**b**) with a two channel detection based on the DoA method. LP: Linear Polarizer. PEM: Photoelastic Modulator. PSA: Polarization States Analyzer. LA: Lock-in Amplifier. BS: Beamsplitter. D: Detector.

In earlier works, numerous CIDS setups only used one PEM in the PSG block and analyzed the intensities with an LP oriented orthogonally to the excited polarized light [77,78,79] as presented in Figure 2.

Using the Stokes–Mueller formalism, it is shown that the differential circular polarized light, coupled with a demodulation detection scheme from a Lock-in Amplifier (LA) at the reference frequency of the PEM, can analyze the differential intensity between the right and left circular polarization states in a few microseconds, allowing extracting the CD/CIDS amplitude in a fast and robust way [80,81]. This simple configuration gives access to only a few elements of the MM, especially $m_{03}$. Furthermore, by rotating the optical axes of the polarizing optics, the number of elements available can be increased, though this results in a longer acquisition time. To improve the acquisition speed, one general method in polarimetry is called Division of Amplitudes (DoA), and it consists of replacing the second linear polarizer by polarized beamsplitters and measuring the complete polarization state of light simultaneously using multiple photodetectors [82,83], as shown in Figure 2b. The drawback of this configuration is the requirement of a precise alignment and an important calibration step, leading to difficulties in obtaining a versatile and compact detection module in terms of light parameters.

**Figure 3:**
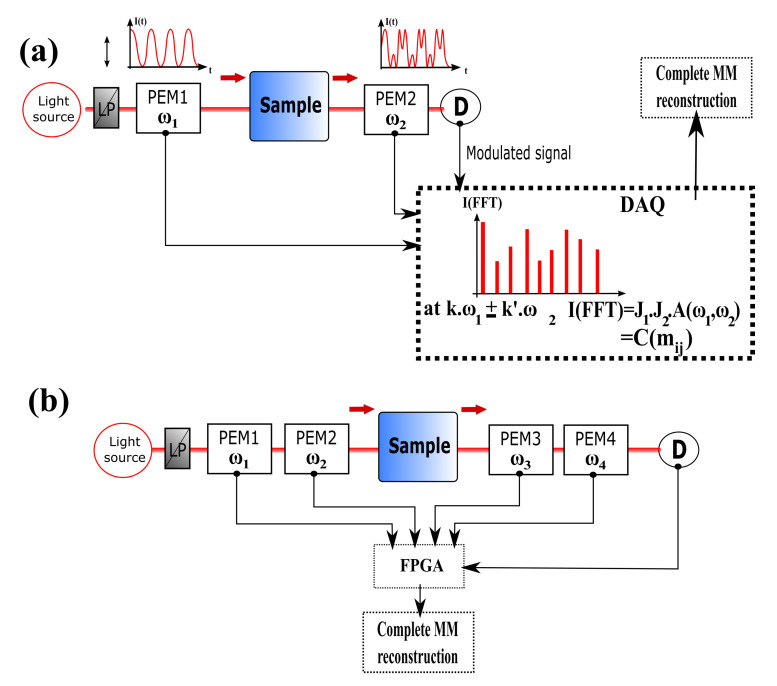
Block diagram of a general complete MM polarimeter using (**a**) two PEMs and (**b**) four PEMs with a single detection coupled with an electronic synchronization. The output intensity temporal spectrum exhibits multiple frequencies $k.\omega_{1}$ and $k^{\prime }.\omega_{2}$, expressed as a mathematical product between Fourier amplitudes $A(\omega_{1},\omega_{2})$ and the Bessel functions $J_{1}$ and $J_{2}$, leading to the combination of the MM elements ($m_{ij}$). The resulting set of equations allow recovering the full MM. LP: Linear Polarizer. PEM: Photoelastic Modulator. LA: Lock-in Amplifier. D: Detector. DAQ: Data Acquisition board. FPGA: field-programmable gate array.

Another solution to improve the acquisition speed is adding a second PEM in the PSA part, phase synchronized with the first one [84], as suggested in Figure 3.

In this method, the common approach is to choose two PEMs at two different working frequencies. This results in an increase of the independent Fourier harmonics, giving access to more equations, therefore more MM elements simultaneously. If the detection system is sensitive enough, all the Mueller coefficients can be obtained in 1 ms [85,86]. Adding a second PEM allows extracting not only the CD signal, but also the LD and the LB and CB [87,88], leading to a better understanding of the sample under illumination. A final upgrade of this technique has been proposed in recent works by adding a third [89] and a fourth PEM [90,91,92]. The main advantage brought by adding a fourth PEM in the CD setup (two for both the PSG and PSA) is that all the elements of the MM can be retrieved without any mechanical moving parts. This optimization of the MM polarimeter, coupled with a triggered synchronization through an FPGA card is a step forward, allowing real-time or snapshot acquisition. Thus, this experimental approach is more suitable for studying ultrafast conformational changes in biopolymers [93,94,95]. In Table 2, we resume the capabilities achieved from the different configurations by using the PEM technology.

## 4. CIDS Microscopy Configuration

### 4.1. Single Point Measurements

**Figure 4:**
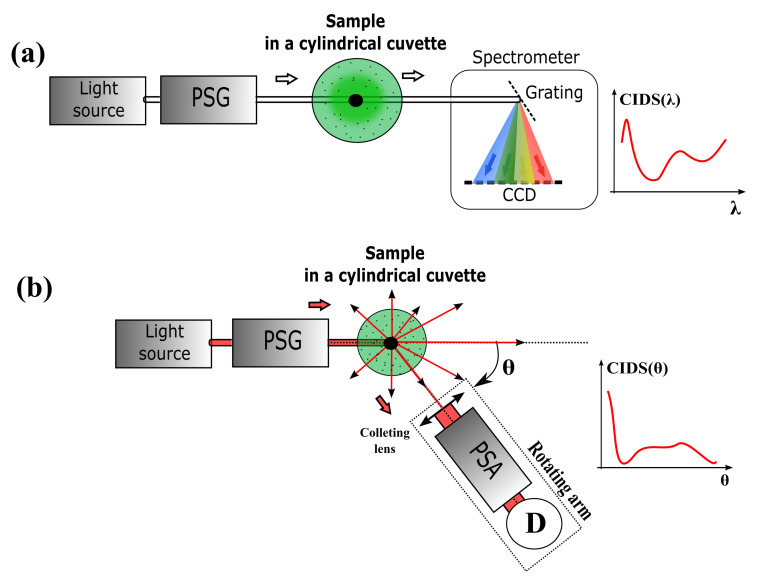
Block diagram of general CIDS experimental setups for (**a**) spectral and (**b**) angular measurements. PSG: Polarization States Generator. PSA: Polarization States Analyzer. θ: Scattering angle. LA: Lock-in Amplifier. D: Detector.

In early works, the CIDS experiments have been performed from samples in suspension of the desired particle or organism inside aqueous solutions. Thus, the signal is derived from the average scattering inside the whole illuminated volume, as shown in Figure 4.

The common measurements for the CD experiments are based on measuring the absorption spectrum of the molecules, giving access to the information of the conformation and handedness at the nanoscale. For this reason, CIDS experiments have been developed by simply modifying commercial spectrometers in order to increase the SNR of the already low scattering signal [96]. However, the angular dependence of the CIDS signal imposes developing new architectures that are able to detect the scattered beam at any angle around the sample. From the first demonstration of the circular polarized light microscope, the CD and more particularly CIDS signal have been used to study biological macromolecules aggregates [32]. With time, it developed an application in the study of various kinds of more complex biological samples like viruses [35], erythrocytes [97], and DNA aggregates [98].

In the case of the spectral measurements in Figure 4a, the detector averages over a smaller angle usually at or close to $0^{\circ }$ of incidence. The information comes from the spectral response, and the light impinges the fingerprint of the macromolecules under illumination. The typical use is identifying different microorganisms from the absorption spectrum of the CIDS. This method has shown the ability of CIDS to differentiate flu viruses and different kinds of live bacteria by using wavelengths between 300 nm and 700 nm [99] or even the diameter of the microorganism population in mixed suspension medium [79]. All microorganisms present a specific CIDS imprint that can be used to identify them in a fast way. The spectral discrimination has been used on Chinese hamster cells to determine the cellular phase by comparing general CD and CIDS signals in different spectral regions [42]. At different cell phases, the nucleus is completely rearranged to transcript the genetic information. It results that the CIDS fingerprint of the DNA could be blurred by the other nucleus components, such as the cytoskeleton or the microtubule structures. CD/CIDS spectroscopy has been demonstrated as an interesting tool in the study of photosynthesis and pigment systems in plants to understand the interactions in pigment-protein complexes [100]. Psi-type (polymer and salt-induced) aggregates like the ones present in chlorophyll and chromophores, as well as DNA were studied and shown to present CIDS outside the absorption bands, which allowed better understanding the energetic bonds in the nucleus [101,102]. The footprint of spinach chloroplasts was used to determine the presence of chiral aggregates inside of the chloroplasts themselves and to determine the radius and pitch of such features by comparing results with simulations [103]. A recent application of CD for characterizing the absorption/scattering spectrum of the chloroplast is the remote identification of such macromolecules in vegetation up to distances of a few kilometers [104].

In the angular measurements in Figure 4b, the setup presents a rotating arm in the sample holder upon which the PSA is mounted. In this way, the signal can be acquired from different angles around the sample, ideally only limited by the mechanical constraints of the components. Experimentally, the condensation of DNA is artificially induced by a large variety of processes, such as by polymers, salts, and H5 histones, in alcoholic solution [98], or simply by dehydration [96]. This produces local compaction of DNA and forms microscopic aggregates, which is the reason DNA is studied in the liquid-crystalline phase in concentrated aqueous solution [105]. Diaspro et al. compared different angular CIDS measurements from several chromatin extraction protocols induced by the arrangement of nucleosomes from rat hepatocytes to decide which method was the closest to preserving in vivo chromatin organization [37,106]. This approach was completed by measurements from the differential calorimetry method [107]. However, it was shown that the CIDS signal is strongly dependent on the extraction protocol of the chromatin, which results in difficulties for interpreting the DNA compaction level. This is the reason that most of the applications for interpreting the angular spectrum of the CIDS are theoretical. Still, they lead to a better understanding in the field of complex nucleic acid organization [31,51,77].

### 4.2. CIDS Optical Imaging Microscopy

MM microscopy imaging has been used widely in the field of biomedical applications for the following reasons: (i) it is a label-free, non-invasive method, allowing contrast without any contact and external labeling and a priori knowledge of the sample; (ii) it is an inexpensive and easily designed tool, which can be performed using white light sources or simple laser diodes and CCD cameras and photodiodes.

In the literature, few works have reported full MM imaging of living or fixed single cells [108,109,110,111]. The difficulty in performing microscopy on these small samples is mainly linked to the diffraction limit in the linear optics regime and the difficulty to preserve the polarization in all the Fields Of View (FOVs) using microscope objectives. Indeed, if the light is focused or collected using a high numerical aperture objective, it may cause polarization distortions [112]. In this case, the polarization is not preserved in all the FOVs at the PSF plane, resulting in a strong depolarization from a spatial averaging of the electric field at the sample plane [113]. One solution to take into account the effect of a tightly focused beam could be introducing new optical devices such as phase plates or vortex plates to compensate for the polarization in all the FOVs [114,115]. Another solution is to describe the polarization in 3D by converting the MM into Hermitian matrices [116], leading to 81 independent MM elements, although it could be reduced to a more manageable 17 MM, only one more than in the 2D MM case [117]. The 2D mapping of the optical activity of the sample through a circular differential imaging configuration was discussed in detailed first by Keller et al. [118]. It has been shown that the circular differential imaging method exhibits contrast from the difference in the optical activity from the surrounded molecules, which is directly correlated to the sample fingerprint without additional staining. The main advantage is the CIDS sign and magnitude of each chiral group is characteristic of structural properties that could be too small to be resolved by conventional microscopy methods. This is a crucial point of this method for structures that present changes as a function of the time or the environment, such as the folding and unfolding chromatin structure. Because CIDS is angularly dependent, the apparent optical activity of each chiral group in the sample is exhibited differently as a function of the excitation light angle and its wavelength. Additionally, two configurations have been considered; (i) in reflection or dark-field imaging and (ii) in transmission or bright-field imaging. It has been shown that the circular reflection differential imaging configuration tends to be more sensitive to large chiral groups with dimensions similar to the wavelength of illumination, while the transmitted configuration is more sensitive to the short-range molecular order. In a series of articles published in the 1980s, this theoretical background for the full MM imaging configuration was completed [119,120,121] using Born approximation at the first and higher orders. It has been shown that the interpretation of the differential circular dichroism images for chiral samples is strongly correlated to the thickness and the degree of anisotropy of the sample [52], leading to neglecting almost all the $m_{ij}$ elements except the ones describing CIDS (i.e., $m_{03}$ or $m_{23}$). Thus, the cross-interaction between the chiral groups affects the optical sectioning pixel-by-pixel, solved using optical scanning confocal microscopy [122], and contributes to polluting the CIDS signal by artifacts in the Point Spread Function (PSF) volume. These simulations have been validated later through experiments for chloroplast organization [123].

**Figure 5:**
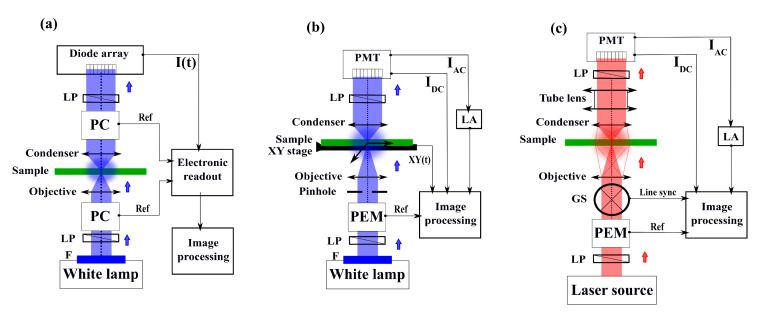
Block diagram of different optical microscope architectures allowing the imaging of the pixel-by-pixel CIDS signal. (**a**) Optical microscope in wide-field configuration using two Pockels cells, inspired by [124,125]. (**b**) Optical microscope using one photoelastic modulator synchronized with a lock-in amplifier and an XY translating sample holder, inspired by [100,126,127]. (**c**) Optical scanning microscope using a photoelastic modulator synchronized with a lock-in amplifier, inspired by [128,129,130]. PC: Pockels Cell. PEM: Photoelastic Modulator. F: monochromatic filter. LP: Linear Polarizer. LA: Lock-in Amplifier. PMT: Photo-multiplier tube. GS: galvanometric scanner.

We summarize the different optical microscopy configurations developed for imaging CIDS pixel-by-pixel in Figure 5.

The first experimental CD/CIDS microscope reported in the literature was proposed by Mickols et al. [124] and is presented in Figure 5a. The idea was to modify an already existing CD microspectrophotometer, using its monochromatic light source and the diode array line-scan detector. Then, the setup was built in the same way as the single point CIDS setups, by adding two Pockels cells in the PSG and PSA optical blocks. Thus, the two Pockels cells were electronically synchronized with the linear diode array to integrate the light over each half cycle of the modulation and form the final image. In this method, the intensity signal was demodulated directly electronically without any use of LA. This was applied first to red blood cells and Chinese hamster ovary cells and then to oxygenated and deoxygenated sickle erythrocytes at 415 nm excitation light [125]. Here, it was first demonstrated that a modification in the concentration and conformation of DNA-based polymers results in a drastic change in CIDS signal. An alternative experimental design of this work was proposed [126], shown in Figure 5b. This new approach became more similar to what can be found for modern experiments by using a lock-in detection. By coupling the advantage of the LA with high quantum efficiency photocathode measurement, the result is a drastic improvement in the SNR, lower power requirements, and fast response (30 ms per pixel). All these changes showed the ability to image and characterize individually red blood sickle cells. Alternative setups based on the same approach of modulation/demodulation of the circularly polarized light were proposed by adding a confocal mode to the microscope. This results in an improvement in the optical resolution giving access to the CIDS signal at the nanoscale in the imaging mode, overcoming artificially the diffraction limit, but sacrificing the acquisition speed by translating point-by point the sample. This allowed for the first time studies for DNA/RNA imaging for different cell cycles or transcriptional activation in live primary spermatocytes of Drosophila [127,131]. Similar setups have highlighted that the sign of the CIDS signal could be changed depending on the buffer of the sample, by mediums in suspension or fixed for the thylakoid membranes of chloroplasts [100,132]. These important results proved that the potential polarimetric artifacts observed with this method could be induced by the superposition of local spectra of opposite sign coming from a mixture of long-range chiral domains in the PSF volume.

**Figure 6:**
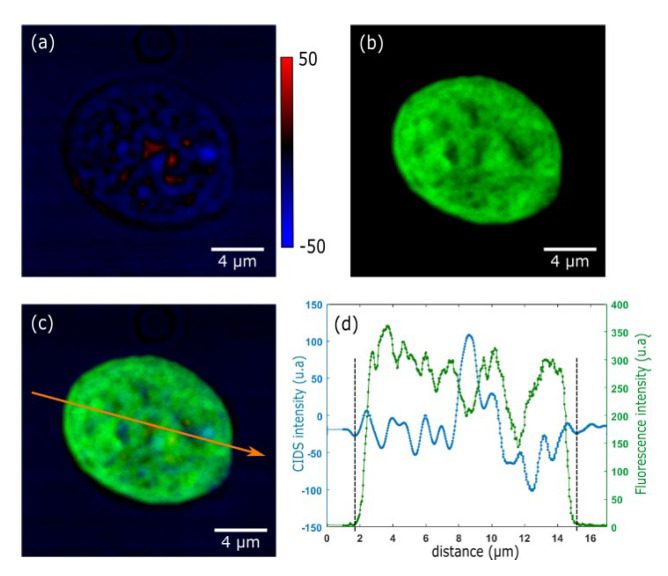
(**a**) Normalized CIDS image of an isolated HEK nucleus after extraction. (**b**) Fluorescence image of the same isolated HEK nucleus labeled with Hoechst. (**c**) Merge of images (**a**,**b**). (**d**) Intensity plot from the orange arrow in (**c**). The blue plot is the intensity profile from the CIDS image (**a**), and the green plot is the Hoechst profile from Image 4. (**b**). The dashed lines indicate the estimated area of the nucleus. Reproduced with permission from [130].

The latest improvement in this research topic comes from the development of the first optical scanning polarization-resolved microscope dedicated to CIDS imaging [128] presented in Figure 5c. It provides a faster acquisition time (few microseconds per pixel) and results in a more robust and sensitive approach. Here, the PEMs are simply placed before and after the microscope body, composed briefly by a tube lens, the galvanometers scanning head, the objective, and the condenser. The demodulated signal, collected by a single point detector, is synchronized with the pixel clock of the scanning head, making possible the reconstruction of the Fourier harmonics of the reference signal of the PEM pixel-by-pixel. This was first demonstrated for chiral materials in a very thin film (tens of Angstroms) at high resolution by overcoming the diffraction limit through the sample. Due to the flexibility of this method and its local sensitivity to optical anisotropy at the nanoscale level, CD/CIDS scanning microscopy has been demonstrated recently as an important tool for label-free imaging in a large range of applications such as for metamaterials characterization [133,134,135,136,137] or chiral polymers organization under certain constraints [129,138,139]. Finally, the versatility of this method has shown its potentiality for multimodal imaging of chromatin characterization proposed recently by our group [130]. We implemented a CIDS imaging modality into a commercial scanning confocal fluorescence microscope in order to observe isolated HEK cell nuclei that were marked with Hoechst 33342, a chromatin binding fluorophore. The idea of observing the cell nucleus with both modalities is to guarantee the imaging of only the fingerprint of the chromatin, independently of residual polarimetric artifacts. By coupling the well-known fluorescence response with our approach, we aimed at proving the validity of CIDS as an imaging mechanism for chromatin-DNA compaction, as shown in Figure 6.

Figure 6a shows the CIDS image, while Figure 6b shows the fluorescence and Figure 6c the merged image. The line profile corresponding to the golden arrow in Figure 6c is shown in Figure 6d for both modalities, in green for fluorescence and blue for CIDS, respectively. As can be seen, the first main result is the correspondence of the edges of the CIDS signal with the ones of the fluorescence signal, confirming that the CIDS signal is produced in the volume of the nucleus, where the chiral organization of chromatin is present. Furthermore, double peaked structures can be noticed in the CIDS signal at the edges of the nucleus corresponding to the heterochromatin, a region transcriptionally inactive where the compaction is higher.

**Figure 7:**
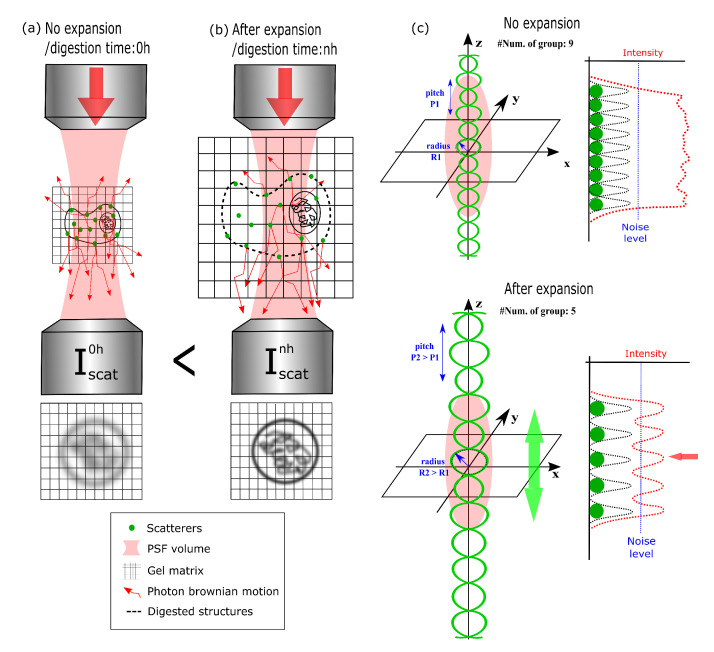
Schematic principle of the scattering process into the PSF volume (**a**) without digestion and (**b**) after n hours of digestion. (**c**) Principle of the SNR decreasing and imaging contrast quality improvement after the expansion process. Reproduced with permission from [140].

Thus, in a following work, we utilized the same technique on whole HEK cells (no nucleus extraction) in conjunction with Expansion Microscopy (ExM) in an approach we named ExCIDS [140]. The ideas behind this method are explained in Figure 7.

**Figure 8:**
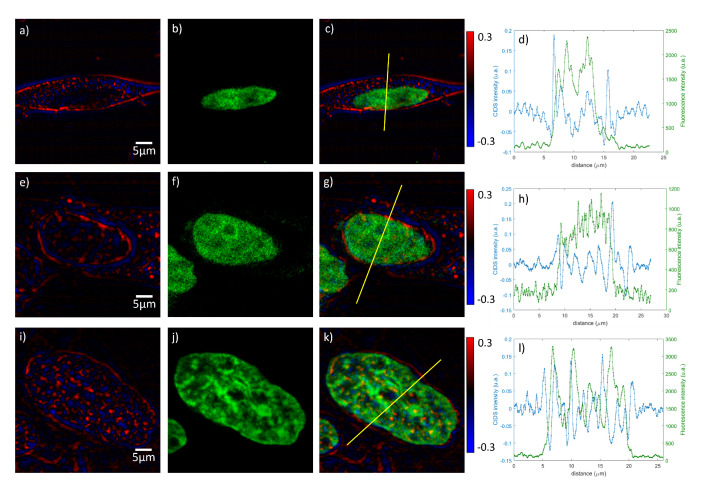
Images of HEK cells labeled with Hoechst in CIDS (**a**,**e**,**i**) and fluorescence (**b**,**f**,**j**) modalities. A merged image of both modalities is presented in (**c**,**g**,**k**), and a line profile relative to the yellow line in those pictures is shown in (**d**,**h**,**l**). Here, CIDS is represented in blue, while the fluorescence signal is represented in green. Reproduced with permission from [140].

The sample before the expansion process is shown Figure 7a. The biological material belonging to the structures outside the chromatin-DNA, like the cellular membrane and cytoplasm, causes scattering of the light that reduces the ability to resolve the chiral group structures inside the nucleus and introduces the signal outside of it. After expansion and digestion in Figure 7b, the quantity and density of biological structures outside the nucleus is lowered, leading to an increase in contrast of the CIDS signal by reducing the scatters. As shown in Figure 7c,d, this has an effect also on the chiral structures of the chromatin-DNA. When the expansion factor increases, the number of chiral structures inside the PSF becomes lower. After a certain point, this number will be too low and the signal will be too close to the noise level, putting a limit on the sample expansion process while keeping a measurable CIDS SNR. Indeed, this has been shown in the paper, where 4× expansion led to loss of CIDS signal, and 2× was deemed the ideal expansion factor for CIDS imaging. As a result, in Figure 8, we show the CIDS and fluorescence imaging results for whole cells before (Figure 8a–d) expansion and at a 2× expansion factor after 4 h (Figure 8e–h) and overnight (Figure 8i–l) digestion and expansion.

As can be seen in the CIDS images Figure 8a,e,i, the CIDS signal present outside the nucleus gradually disappears with the increase of digestion time due to a digestion of the biological components of the cell, while keeping a good SNR inside the nucleus itself.

The next step of this work is to combine additional label-free modalities, such as Ptychography [141] or SHG (Second Harmonic Generation) with multiple fluorescence modalities, such as Fluorescence-lifetime imaging microscopy (FLIM), STimulated Emission Depletion (STED), or multispectral fluorescence channels imaging with CIDS to have a full and deep understanding of the chromatin at different DNA compactions and cell cycles [142].

## 5. Conclusions

The CIDS technique is an interesting label-free approach for understanding the molecular organization at the nanoscale, demonstrating its potentiality in the field of chromatin structural characterization. This method has several advantages over other traditional optical imaging modalities such as being a versatile and flexible tool, with low complexity and low cost. In this review, we presented the earlier and the most advanced models that have proven their accordance to experimental data. We showed that the experimental architectures are inspired by Mueller–Stokes setups, and they are simple to handle. However, a high sensitivity in the polarized detected light is required to track fast molecular changes in the biopolymer structure. It follows that switching this simple configuration into an imaging system is not straightforward, and a different source of artifacts could be measured. It is induced mostly by the limitation of such a linear optical technique to measure the average CIDS signal through the entire PSF volume, without any spatial sectioning. Thanks to the recent advances and demonstrations offered by the upgrade of the optical scanning microscope, it is easy to imagine 3D real-time measurement in the polarization-based imaging research field.

## Figures and Tables

**Table 1:** Non-inclusive report of the main performances and capabilities of the commonly used polarization modulation devices in CIDS setups, meaning simple polarizing optics (assembled in rotors), Pockels Cell (PC), and Photoelastic Modulator (PEM).

	Speed	Spectral Range	Advantages	Disadvantages
Rotors	∼ Hz	Wide	Easy and simple Inexpensive	Precise alignment Electronic controls
PC	DC—1 GHz	200 nm to 5 μm	High optical quality Very fast modulation Compactness	Temperature control Residual stress Mathematical model Expensive
PEM	20–84 kHz	170 nm to 19 μm	High optical quality High power handling Large acceptance angle Compactness	Mathematical model Expensive

**Table 2:** Main characteristics of the single and multiple PEM-based technology experiments.

	Number of Element	Speed	Advantages
1 PEM	4	100 ms	Simple and easy
2 PEMs	8	10 ms	No lock-in detection needed
3 PEMs and 4 PEMs	16	1 ms	No lock-in detection needed No moving parts Wide range of applications

## Abbreviations

The following abbreviations are used in this manuscript:

CD	Circular Dichroism
CIDS	Circular Intensity Differential Scattering
2D	Two-Dimensional
3D	Three-Dimensional
MM	Mueller Matrix
DDA	Discrete Dipole Approximation
SNR	Signal to Noise Ratio
PSG	Polarization States Generator
PSA	Polarization States Analyzer
PSD	polarization States Detector
DOP	Degree Of Polarization
DOLP	Degree Of Linear Polarization
DOCP	Degree Of Circular Polarization
PSF	Point Spread Function
LP	Linear Polarizer
HWP	Half-Waveplate
QWP	Quarter-Waveplate
PEM	Photoelastic Modulator
PC	Pockels Cell
LA	Lock-in Amplifier
DoA	Division of Amplitudes
LD	Linear Dichroism
LB	Linear Birefringence
CB	Circular Birefringence
FOV	Field Of View
ExM	Expansion Microscopy
SHG	Second Harmonic Generation
FLIM	Fluorescence-lifetime imaging microscopy
STED	STimulated Emission Depletion
